# Fine mapping of interactions between eEF1α protein and 3′UTR of metallothionein-1 mRNA

**DOI:** 10.1016/j.bbrc.2009.05.146

**Published:** 2009-08-14

**Authors:** Kunbo Fan, Zofia M.A. Chrzanowska-Lightowlers, John E. Hesketh

**Affiliations:** a Institute for Cell and Molecular Biosciences, Newcastle University, The Medical School, Framlington Place, Newcastle upon Tyne NE2 4HH, UK; b Institute for Ageing and Health, Newcastle University, The Medical School, Framlington Place, Newcastle upon Tyne NE2 4HH, UK

**Keywords:** Metallothionein-1, Perinuclear mRNA localisation, 3′Untranslated region, Elongation factor 1α, RNA binding protein, RNA secondary structure, Histidine-tRNA ligase

## Abstract

The localization of metallothionein-1 (*MT-1*) mRNA to the perinuclear cytoskeleton is determined by a signal in the 3′untranslated region (3′UTR) and *trans*-acting binding proteins. The present study carried out detailed mapping of this signal and further characterized the binding to elongation factor 1 alpha (eEF1α) and other interacting proteins. Electrophoresis mobility shift assays demonstrated that shortening of a stem region proximal to nucleotides 66–76 abrogated binding. Full length recombinant rat eEF1α, and independently domains I and III, formed complexes with the mRNA. Proteins binding to biotinylated *MT-1* 3′UTR sequences were isolated using RNA-affinity techniques, and mass spectrometry identified histidine-tRNA ligase as one of the major *MT-1* 3′UTR binding proteins. We conclude that a 5-bp internal stem in the *MT-1* 3′UTR is critical for binding of eEF1α and histidine-tRNA ligase, and that binding of eEF1α is facilitated through domains I and III.

## Introduction

In eukaryotic cells the defined distribution of specific mRNA species to different regions of the cytosol provides a mechanism for localized synthesis of proteins close to their site of action [1,2]. In fibroblasts, the transcripts encoding β-actin are transported to the cell periphery [3] whereas, in contrast, *c-myc*, *c-fos*, and *metallothionein-1* (*MT-1*) mRNAs are retained around the nucleus [4–6]. This perinuclear localization of *MT-1* mRNA is essential for nuclear localization of MT-1 protein in S phase and for the ability of MT-1 to limit DNA damage [7,8]. Thus, perinuclear localization of *MT-1* mRNA appears to facilitate the subsequent import of the protein into the nucleus under appropriate conditions. Nuclear localization of MT-1 may, therefore, be physiologically important during transition from G_1_ to S phase, following cell stress induced by cytotoxic agents or following stimulation by nitric oxide [9–11].

**Fig. 1:**
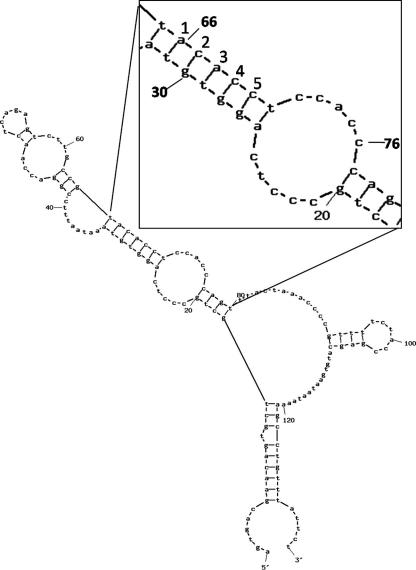
Predicted secondary structure of *MT-1* 3′UTR. The secondary structure was predicted using Mfold (http://mobyle.pasteur.fr/cgi-bin/portal.py?form=mfold). The region studied in this work (magnified to increase clarity) was the stem formed by nt 26–31 and 66–70 and the CACC repeat between nt 66 and 76.

In general mRNA localization is brought about by *cis*-acting signals within the 3′untranslated region (3′UTR) that interact with RNA-binding proteins [1,2,12]. Localized mRNAs appear to be transported in RNA granules where they are associated with several proteins [13,14]. The nature of such complexes remains to be defined. In the case of perinuclear mRNAs neither the precise nature of the signal within the 3′UTR nor the nature of the binding complex have been characterised. However, for *MT-1* mRNA, there is evidence that the region of the 3′UTR between nucleotides (nt) 45 and 76, particularly nt 66–76, is required for localization [15]. This region contains CACC repeats and interestingly such repeats have been implicated in other localization signals [16,17]. Data derived from a combination of bioinformatic folding predictions and chemical cleavages indicate that this region is part of an internal stem-loop (see Fig. 1) and initial mutagenesis studies suggested that the secondary structure is important for localization [6]. This earlier study indicated that elongation factor 1 alpha (eEF1α) binds to this region [6], and furthermore eEF1α has also been reported to bind to the localization signal of *β-actin* mRNA [18]. To date neither the relative importance of the CACC repeat in combination with the stem-loop structure nor the domains of eEF1α required for this binding have been fully elucidated. Moreover, it is not known which proteins, in addition to eEF1α, are present as part of the localization complex.

This work carried out a systematic study of the role of the internal stem-loop region of the 3′UTR and the domains of eEF1α in *MT-1* transcript–protein complex formation, and identified a further protein component of the complex. Our approach has been to combine mutagenesis, over-expression and purification of recombinant eEF1α and RNA–protein binding assays to characterize the *MT-1*-3′UTR–eEF1α interaction. RNA affinity and proteomic analysis were employed to identify protein components of the complex.

## Material and methods

*Gene constructs.* Mutant constructs (deletions or substitutions) of *MT-1* 3′UTR were made by site-directed mutagenesis using as template the pcDNA4/Hismax-TOPO plasmid containing the rat *MT-1* sequence corresponding to the 3′UTR (pc*MTfull*; 6) and following the manufacturer’s instructions (Stratagene, Quikchange^®^). The primers used for the mutagenesis are listed in Table 1. All constructs were verified by sequencing using the T7 forward and BGH reverse primers.

*In vitro transcription of radiolabelled and unlabelled RNAs.* Templates for transcription of rat *MT-1* 3′UTR nucleotides 1–111 and the various mutants were generated by PCR from pc*MTfull*, or the appropriate mutant construct, with the following primers;- T7 Forward 5′-TAATACGACTCACTATAGGAGTGACGAACAGTGCTGCTG-3′ containing the promoter sequence (underlined) and Reverse 5′-CACATGCTCGGTAGAAAACGG-3′. PCR products were purified using QIAquick columns (Qiagen). For electrophoretic mobility-shift assays RNA corresponding to nucleotides 1–111 of the *MT-1* 3′UTR (*MT-111*) was synthesized incorporating radiolabelled [α-^32^P]CTP (GE Healthcare) using the MAXIscript^®^ kit (Ambion, Applied Biosystems). The synthesized *MT-111* mRNA was purified by phenol–chloroform extraction, and ethanol precipitated. Unlabelled RNA or transcripts incorporating biotin-16UTP (Roche) were produced using the MEGAshortscript kit (Ambion) and quantified spectrophotometrically.

*Electrophoretic mobility-shift assays (EMSA).* Chinese hamster ovary (CHO) cells were grown in Ham’s F-12 medium supplemented with 10% foetal calf serum, in an atmosphere of 5% CO_2_ at 37 °C. Cells were grown to ∼90% confluence and cytoplasmic protein extracts were prepared following the method of Behar et al. [6,19]. Reactions were carried out with 2 μg protein extract and 12 fmoles of ^32^P-labelled *MT-111* RNA (denatured at 70 °C and refolded slowly) in dilution buffer (40 mM NaCl, 5 mM MgCl_2_, 30 mM Tris–HCl pH 7.6, 2 mM DTT, EDTA-free protease inhibitor cocktail (Roche)) in a total volume of 8 μl at 22 °C for 15 min. Following the binding reaction, 40 units (U) of RNase T1 were added and incubation continued for 15 min. 2 μl of 20% (w/v) Ficoll was then added and complexes were separated by electrophoresis at 4 °C for 2 h at 20 V/cm through 5% (w/v) non-denaturing polyacrylamide gels. Mutant, non-radiolabelled transcripts were added as competitors to the binding reaction concurrently with the radiolabelled *MT-111* transcripts. Gels were dried and radioactivity visualized by autoradiography.

*Preparation of recombinant elongation factor 1 alpha (eEF1α).* N-terminal GST fusion proteins of rat eEF1α were over-expressed as full length or sub-domains I, II or III in BL-21 Rosetta cells by induction with 1 mM IPTG at 37 °C for 2.5 h (GST-eEF1α domain II and pure GST protein) or 0.2 mM IPTG at 16 °C for 16 h (GST-eEF1α full length, domains I and III). All the pGEX vectors were kindly donated by Gang Liu (Albert Einstein College of Medicine). Recombinant GST protein alone was also purified and used as a control. Cell pellets were re-suspended in pre-cooled resuspension buffer (1× PBS, 5 mM β-mercaptoethanol), disrupted by sonication and the suspension centrifuged at 30,000*g* at 4 °C for 30 min. The supernatant fluid was filtered (0.2 μm) and incubated with glutathione Sepharose™ 4B (GE Healthcare). After washing, bound proteins were eluted with 10 mM reduced glutathione, 50 mM Tris pH 8.0, 50 mM NaCl, 10 mM β-mercaptoethanol. The purity of eluted protein was examined by SDS–PAGE and aliquots stored with 15% glycerol at −80 °C.

*Isolation of interacting proteins using paramagnetic beads.* Biotinylated *MT-111* RNA (20 μg) was heated at 70 °C for 5 min, 40 °C for 20 min and left to cool to room temperature to facilitate native folding. The MagneSphere streptavidin-coated paramagnetic particles (SA-PMP, Promega, 0.6 ml) were pre-treated with 0.1 ml 0.5× SSC buffer containing 100 μg BSA and 100 μg yeast tRNA at room temperature for 1 h. Beads were washed twice (0.5× SSC) and incubated with 20 μg biotinylated *MT-111* mRNA in 0.3 ml of 0.5× SSC buffer for 10 min at room temperature. Unbound mRNA was removed by extensive washes (0.5× SSC buffer). Beads were then incubated for 1 h with 1 mg CHO S100 cell extract in dilution buffer supplemented with 0.5 mg/ml yeast tRNA, 0.2 mg/ml BSA and 800 U/ml RNasin (Promega) in a final volume of 0.5 ml at 4 °C. After incubation, beads were washed 5 times in dilution buffer and resuspended in 25 μl of the same buffer. An aliquot (5 μl) was subjected to 10% SDS–PAGE and the proteins present were visualized by colloidal Coomassie stain (Sigma). Major bands were excised for in-gel trypsin digestion and analysis by matrix-assisted laser desorption/ionization time-of-flight (MALDI-TOF) mass spectrometry (performed by Dr. J. Gray, Institute for Cell and Molecular Biosciences, Newcastle University) using a Voyager DE-STR MALDI-TOF mass spectrometer (Applied Biosystems). Mass spectra were obtained over a 900–4000 Da range and monoisotopic peptide mass fingerprints were assigned and used for databases searches. Identifications were performed using the Mascot search engine program (Matrix Science Ltd, London).

## Results and discussion

### Signal elements that are critical for RNA binding

**Fig. 2:**
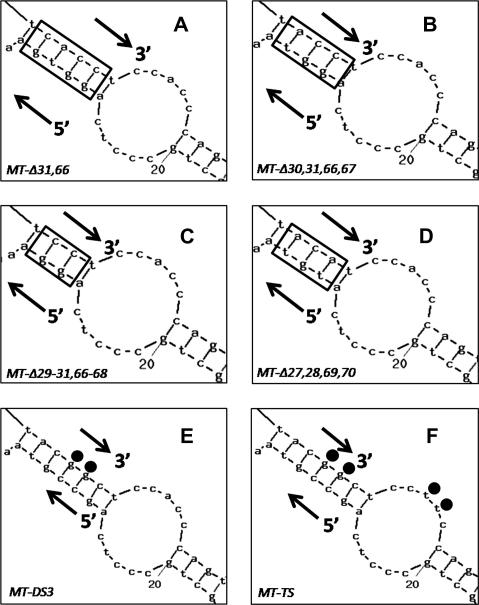
Predicted secondary structure for mutant *MT-1* 3′UTR sequences. The secondary structure of the various mutant transcripts produced by site-directed mutagenesis was predicted using Mfold (http://mobyle.pasteur.fr/cgi-bin/portal.py?form=mfold). (A–D) Show mutants in which the internal stem is predicted to be shortened; the remaining stem is indicated by the rectangle in each case: pc*MT-Δ31,66* has 1 bp deleted (A), pc*MT-Δ30,31,66,67* has 2 bp deleted (B), pc*MT-Δ29–31,66–68* 3 bp deleted (C) and in pc*MT-Δ27,28,69,70* the 2 bp close to the loop region are deleted (D). (E and F) Show mutants in which the CACC repeat sequence is altered but base pairing in the stem maintained: pcMT-DS3 had two nucleotides in the stem region CACC sequence substituted (E) and pcMT-TS had both CACC sequences, one in the stem region and one in the loop, interrupted by nucleotide substitution (F).

A combination of chemical cleavage analysis and secondary structure prediction of the *MT-1* 3′UTR has implicated a CACC repeat within a region that contains a internal stem-loop region as being a critical element required for the localization of *MT-1* mRNA [6]. The subcellular distribution of the transcript is facilitated by interaction with protein(s) binding to its 3′UTR. To characterise the significance of the CACC repeat and the stem-loop in these protein interactions, a series of mutant *MT-1* transcripts were generated by site-directed mutagenesis. The internal stem is predicted to contain 5 bp (nt 27–31 interacting with 66–70) as shown in Fig. 1. Based on this predicted structure four mutants were made in which, as shown in Fig. 2, the deletions were predicted by Mfold to shorten the stem by one, two or three base pairing nucleotides. Thus, the mutant pc*MT-Δ31,66* was predicted to produce transcripts with a stem shortened by 1 bp: similarly the stem in *MT-Δ30,31,66,67* was 2 bp shorter, and *MT-Δ29–31,66–68* by three, whilst *MT-Δ27,28,69,70* was predicted to remove 2 bp positioned proximal to the loop region. Two further mutants were made that were predicted to maintain a 5-bp stem but in which the CACC sequence in either the stem or the adjacent loop had been replaced. In *MT-DS3* the CACC sequence in the stem region (nt 67–70) was substituted with CGGC and mutant *MT-TS* had the CACC sequences in both the stem region and loop region replaced with CGGC and CTTC, respectively.

**Fig. 3:**
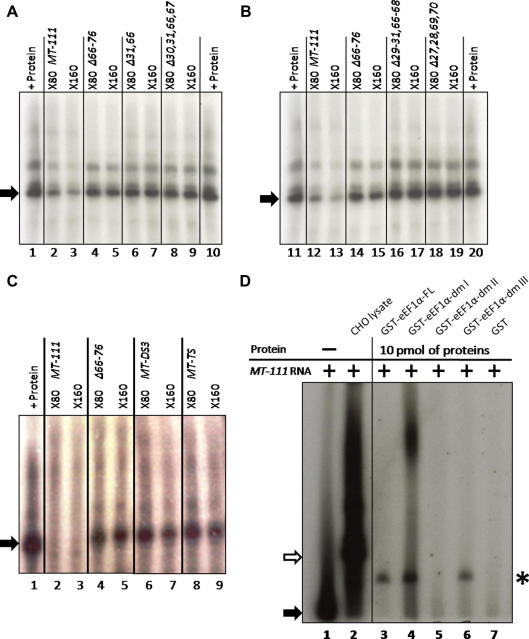
EMSA investigation of binding of CHO cellular proteins and recombinant rat-eEF1α to wildtype and mutant *MT-1* 3′UTR transcripts. (A–C) Show competition EMSA to test binding by CHO cell S100 extracts of *MT-1* 3′UTR mutants containing shortened stem or mutated CACC sequences. The major mRNA–protein complex formed by radiolabelled wildtype *MT-111* mRNA and CHO cell lysate only, without the addition of any competitor, is indicated by the black arrow. The remaining lanes represent reactions that include different non-radiolabelled competitor transcripts at either 80- or 160-fold molar excess. (D) Show EMSA reactions carried out with wildtype *MT-111* transcripts and either CHO cell extracts or recombinant eEF1α proteins. Lane 1 represents *MT-111* probe alone (indicated by black arrow). The major complex formed between CHO cell lysate and *MT-111* RNA is shown in lane 2 (white arrow). Reactions with domains I–III of eEF1α protein are shown in lanes 3–6 (complex formation is indicated by the asterisk) and a control of GST alone in lane 7. Native PAGE was carried out at 20 V/cm for 2 h and complexes detected by autoradiography.

EMSA demonstrated that wildtype *MT-111* transcripts formed a complex with protein(s) in CHO cell S100, as previously described [6,15]. RNase T1 digestions following binding revealed several retarded complexes with a single major complex predominating (Fig. 3). The ability of the mutant transcripts to compete against the wildtype RNA (*MT-111*) for protein binding was assessed by competition EMSA using 80- to 160-fold molar excess of unlabelled mutant RNA species. Unlabelled *MT-111* RNA was used as a positive competitor control and *MT-Δ66–76* transcripts were used as a negative competitor control since the latter has been shown previously not to compete for binding [6]. Competition EMSA was performed with each of the various stem shortened mutants (Fig. 3A and B). In each case the competitor RNA failed to inhibit complex formation between *MT-111* RNA and proteins from the CHO cell S100 fraction. Thus removal of bases 31:67, 30/31:66/67, 29/30/31:66/67/68 or 27/28:69/70 (Fig. 3A and B) resulted in an inability of the mutant transcript to bind to CHO proteins. Similarly, the mutant RNA where the CACC sequence in the stem had been substituted whilst retaining base pairing in the internal stem (*MT-DS3*) also failed to compete with *MT-111* RNA for protein binding (Fig. 3C) Moreover, mutant RNA again retaining the internal stem but with both CACC repeats substituted (*MT-TS*) also failed to compete for binding. Thus, loss of the CACC domains also abrogated binding to CHO cell proteins. Overall, this data extends previous observations by indicating that the length of the stem is a critical feature determining specific protein binding; any shortening of this stem region destroyed the ability to interact with CHO proteins. The CACC sequence within this stem region is essential for binding since its substitution also destroys binding although the internal stem base pairing is retained. Since protein binding to this region has been correlated with mRNA localization [6,15] the data suggest that the internal stem and CACC repeat are critical features of the perinuclear localization signal.

### Binding studies with recombinant eEF1α

Previous studies have suggested that eEF1α in CHO cell extracts binds to the internal stem-loop localization signal in the *MT*-3′UTR [6]. However, it was not identified whether (i) eEF1α can bind RNA independently, (ii) whether eEF1α requires other proteins to successfully generate a binding complex, or (iii) if specific single or multiple domain(s) of eEF1α are responsible for RNA interactions. This study aimed to investigate these questions. Purified recombinant proteins corresponding to the full length eEF1α or independent sub-domains were generated and used in EMSA in conjunction with wildtype *MT-111* transcripts. Incubation of full length recombinant eEF1α with *MT-111* transcripts led to a retardation of the RNA, indicating formation of a complex. GST alone failed to generate this reduced mobility of the transcript and this was also true of domain II. Domains I and III interacted with the RNA as did the full length protein, generating a complex (Fig. 3D). Domain I generated a second complex of slower migration, suggesting that it may bind as a multimer. In comparison, the two major complexes formed between CHO S100 cell extract and the wildtype mRNA migrated more slowly in the gel than the complex formed with recombinant eEF1α. The migration of the complex generated by domains I, III and the full length protein is similar to one of the minor species from the EMSA with CHO cell S100 extract. These data suggest that the full binding complex comprises more than just eEF1α and that both domains I and III are independently capable of binding to *MT-111* transcripts, but that domain II cannot bind.

Previous studies have identified that recombinant eEF1α binds to the 3′UTR of *ß-actin* mRNA [18] and that binding was facilitated by domains I and III. The results presented here also show that domains I and III are required for binding to *MT-1 3*′*UTR*. Interestingly, although these two mRNAs are localized to different regions of the cytosol they are both associated with the cytoskeleton [3,5], and in both cases it has been suggested that the mRNAs are anchored on microfilaments. Since eEF1α is a major actin-binding protein [20] it is possible that it may function to anchor mRNAs to the cytoskeleton. The present data and those of Liu et al. [18] suggest that it is domains I and III of eEF1α that are involved in binding to the mRNA.

### Identification of binding complex proteins from CHO cell extract

**Fig. 4:**
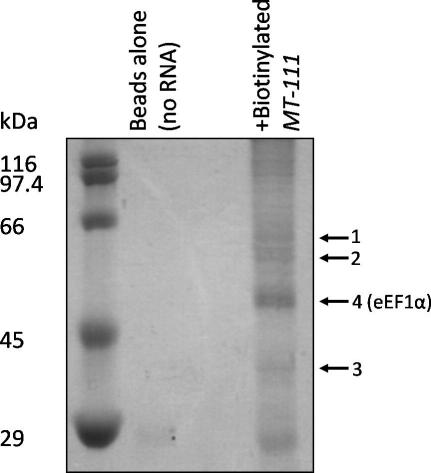
Isolation of proteins binding to biotinylated *MT-111* RNA. Reactions containing CHO cell S100 extract with or without biotinylated *MT-111* were subjected to RNA-affinity chromatography with streptavidin-coated paramagnetic beads. Proteins eluted from the beads were separated by 10% SDS–PAGE. Major protein species are indicated (black arrows); these were excised and subjected to analysis by mass spectrometry.

It was notable by EMSA using recombinant eEF1α that the complex formed was retarded less than the complex formed between the *MT-111* RNA and CHO cell lysate (see Fig. 3D), suggesting that proteins other than eEF1α in the CHO cell lysate may also be involved in complex formation. In order to identify these proteins biotinylated *MT-111* RNA was immobilized on streptavidin beads and used as bait to isolate interacting proteins from a CHO cell lysate. The interacting proteins were eluted and separated by SDS–PAGE. Coomassie staining revealed a number of proteins from the *MT-111* transcript affinity column but not from the control column containing beads alone (Fig. 4). Four major species, with approximate molecular weights of 61, 57, 40 and 50 kDa, were excised from the gel and analysed by mass spectrometry.

The analysis of the 50 kDa band identified it as eEF1α, consolidating previously published data [6]. The protein present in the ∼57 kDa band (band 2 in Fig. 4) was identified as Chinese hamster histidine-tRNA ligase (Mowse score = 61, *p* < 0.05), which with a molecular mass of 57 kDa closely matched the migration by SDS–PAGE. Thus, it appears that *MT-111* transcripts interact directly, or via eEF1α, with histidine-tRNA ligase and are part of an RNP complex that contains several proteins. This finding of a second protein in the complex that has been previously identified to have a different function in protein synthesis, is consistent with the multifunctional nature of proteins that have been found to bind to localization signals [21–24]. It is also consistent with observations that components of the translational apparatus are co-transported with mRNAs [13,14] and that they too have been found to bind to localization signals in mRNAs [23,24]. SDS–PAGE analysis of the eluates from the RNA affinity column suggests that in addition to eEF1α and histidine-tRNA ligase, other proteins may be involved in complex formation. However, analysis by MALDI-TOF of the two further bands (bands 1 and 3 in Fig. 4) failed to reveal any significant match. Hence, further investigation is needed to identify these co-purifying proteins that may form critical members of the binding complex.

In conclusion, we postulate that eEF1α is capable of binding to a specific internal stem-loop region of the 3′UTR of *MT-1* mRNA implicated in perinuclear mRNA localization and hypothesise that histidine-tRNA ligase is also a member of this binding complex. Fine mapping of the critical regions involved in complex formation shows that domains I and III of eEF1α protein are sufficient for recognition of a RNA motif in which both the 5-bp stem and CACC repeat sequence are essential. Although these domains of eEF1α can act independently to facilitate this binding, it is likely that other proteins are present in the RNP complex *in vivo*. Interestingly, eEF1α also binds to the zip-code sequence in actin mRNA and a stem-loop structure in the West Nile virus [3,18,25] and these motifs also contain an ACACC or CAC repeat suggesting that a combination of stem-loop structure and CAC repeat sequence is a conserved motif important in binding of eEF1α to specific transcripts.

## Figures and Tables

**Table 1:** Site-directed mutagenesis primers for generating mutants with shortened stem region or altered CACC repeat motif in *MT-1* 3′UTR.

Mutation	Forward	Reverse
*Primers listed below are for making mutants with shortened stem region of MT-1 3′UTR*		
Δ31	5′-gccctcaggtgaaataatttccg-3′	5′-cggaaattatttcacctgagggc-3′
Δ66	5′-gagtcttgccgtcacctccaccc-3′	5′-gggtggaggtgacggcaagactc-3′
Δ30,31	5′-gctgccctcaggtaaataatttccgg3′	5′-ccggaaattatttacctgagggcagc-3′
Δ66,67	5′-gagtcttgccgtacctccacccag-3′	5′-ctgggtggaggtacggcaagactc-3′
Δ29–31	5′-gctgccctcaggaaataatttccgg-3′	5′-ccggaaattatttcctgagggcagc-3′
Δ66–68	5′-gagtcttgccgtcctccacccag-3′	5′-ctgggtggaggacggcaagactc-3′
Δ27,28S1	5′-gctgctgccctcagtgtaaataatttcc-3′	5′-ggaaattatttacactgagggcagcagc-3′
Δ27,28S2	5′-gctgctgccctcatgtaaataatttcc-3′	5′-ggaaattatttacatgagggcagcagc-3′
Δ69,70S1	5′-cttgccgtacactccacccagtttac-3′	5′-gtaaactgggtggagtgtacggcaag-3′
Δ69,70S2	5′-cttgccgtacatccacccagtttac-3′	5′-gtaaactgggtggatgtacggcaag-3′

*Primers listed below are for making mutants with altered CACC repeat motif in MT-1 3′UTR*		
Δ28,29	5′-gtgctgctgccctcagccgtaaataatttccggac-3′	5′-gtccggaaattatttacggctgagggcagcagcac-3′
Δ68,69	5′-cagagtcttgccgtacggctccttccagtttac-3′	5′-gtaaactggaaggagccgtacggcaagactctg-3′
Δ74,75	5′-gtcttgccgtacacctccttccagtttactaaaccccg-3′	5′-cggggtttagtaaactggaaggaggtgtacggcaagac-3′

Each site-directed mutagenesis reaction effected a single nucleotide deletion when producing shortened stem *MT-1* 3′UTR mutants or replacement of two nucleotides when making *MT-1* 3′UTR mutants containing altered CACC repeat motif. The designated removal or substitution of several bases was achieved by sequential PCR with the forward and reverse primers given above.
